# The effects of skin tone, height, and gender on earnings

**DOI:** 10.1371/journal.pone.0190640

**Published:** 2018-01-02

**Authors:** Srikant Devaraj, Narda R. Quigley, Pankaj C. Patel

**Affiliations:** 1 Center for Business and Economic Research, Ball State University, Muncie, Indiana, United States of America; 2 Management and Operations, Villanova University, Villanova, Pennsylvania, United States of America; Universite de Lausanne, SWITZERLAND

## Abstract

Using a theoretical approach grounded in implicit bias and stereotyping theories, this study examines the relationship between observable physical characteristics (skin tone, height, and gender) and earnings, as measured by income. Combining separate streams of research on the influence of these three characteristics, we draw from a sample of 31,356 individual-year observations across 4,340 individuals from the National Longitudinal Study of Youth (NLSY) 1997. We find that skin tone, height, and gender interact such that taller males with darker skin tone attain lower earnings; those educated beyond high school, endowed with higher cognitive ability, and at the higher income level (>75^th^ percentile) had even lower levels of earnings relative to individuals with lighter skin tone. The findings have implications for implicit bias theories, stereotyping, and the human capital literature within the fields of management, applied psychology, and economics.

## Introduction

Recent advances in the literature on stereotyping and implicit bias have increased our understanding of the processes by which observable individual physical characteristics may be perceived by others and may adversely impact organizational decision-making. [1], [2], [3], [4], [5], [6], [7], [8], [9] Prior research has confirmed that individuals with darker skin tone, shorter individuals, and women all experience lower income levels over time, yet no research to our knowledge has examined the potential impact of all three variables at once. The scholarship on intersectionality has begun to examine the impact of the combination/fusion of race, gender, social class, and sexuality on workplace outcomes, [10], [11], [12], [13] but this research stream has not included the examination of skin tone and other observable, physical attributes such as height and weight.

We develop propositions based on implicit bias and stereotyping theories through the lens of dual process models. According to these, individuals may have two distinct modes of information processing—an automatic mode, in which they use simple heuristics to make decisions quickly without a great deal of mental effort, and a systematic mode, in which they process information in a deliberate, more reflective manner. [2] Both types of information processing models may lead to stereotyping biases amongst target groups. [14], [15], [16], [17], [18]. While we acknowledge that we cannot empirically parse out the exact cognitive path through which this occurs, we propose that height and skin tone could lead to stereotype activation and subsequent cognitive bias. Notably, there are no legal protections addressing these two characteristics in the U.S. with respect to workplace discrimination.

This study makes at least three important contributions. First, we focus on skin tone bias as a distinct issue. Racial minorities face more negative workplace outcomes than white individuals; [13], [19], [20] moreover, skin tone bias (i.e., a systematic bias associated with how light/dark an individual’s skin is perceived to be) is pervasive, [21], [22], [23], [24], [25] even within racial and ethnic groups. There is a greater need for the organizational literature to acknowledge and examine skin tone bias further. Second, this study’s combined focus on multiple, observable physical attributes is rare in the organizational literature (the research on height and weight-related employment discrimination is a notable exception; e.g., [26]). Third, we examine a “millennial” sample from the National Longitudinal Survey of Youth (NLSY) 1997, a longitudinal study of individuals in the U.S. born between 1981 and 1985. The 80-million-plus millennials are the most diverse generation to date (60% is considered to be non-Hispanic white, 19% Hispanic, 14% black, 4% Asian, and 3% mixed race; 11% are born to at least one immigrant parent). The study of the extent to which these individuals will face implicit bias, stereotypes, and adverse impact at work is relevant and timely.

Following Kreisman and Rangel [27] and Neal, [28] we include only individuals who identified themselves as either white or black, as these two categories are the most commonly compared groups among inter- and intra- racial literature on demographic and social issues.

## Theoretical development

We start by exploring the extant literature on skin tone, height, and gender.

### Skin tone

We define skin tone bias in keeping with recent work [21], [22], [24], [25] as the tendency to use an individual’s skin tone lightness or darkness to develop behavioral intentions toward that individual. In addition to bias and discrimination associated with race and ethnicity, skin tone bias remains one of the most pervasive issues affecting perception and decision making. [24], [25] Skin tone bias is known to be “pervasive across and within diverse ethnic and racial groups, including Whites, Latinos, and Blacks.” ([21], p. 1)) As noted above, while we acknowledge that race itself is related to workplace outcomes and earnings, in the sense that racial and ethnic minorities face more bias, discrimination, and negative workplace outcomes than white individuals, like Hunter [23] we note that the “two systems of discrimination (race and [skin tone bias]) work in concert… [although they are] distinct” (p. 238). We focus specifically on skin tone in this particular study, while acknowledging that (and controlling for) the category of race/ethnicity, which is an important consideration.

The literature on skin tone bias has been developing for decades in various fields. [23], [29], [30], [31], [32], [33], [34] Sociologists have long considered skin tone bias across the world as a social issue with deeply ingrained historical roots. Hunter ([23], p.239), for example, notes that skin tone bias was apparent as slave owners “typically used skin tone as a dimension of hierarchy on the plantation”; lighter-skinned slaves typically worked in homes, while slave owners assigned darker-skinned slaves to work in the fields. In modern society, sociologists have studied the influence of skin tone bias in a number of decisions, from hiring to marriage. [35], [36], [37], [38], [39] Within the field of economics, the topic of skin tone bias has become more visible relatively recently. Several studies have linked skin tone to income levels, with the overall finding that darker skin tone seems to be negatively related to income. [27], [36], [40], [41] Kreisman and Rangel’s [27] study nests skin tone within race (i.e. black and white), allowing the opportunity to (1) control for inter-group gaps in the labor market between black and white individuals and (2) to assess intra-racial gaps in labor market outcomes among black individuals of varying skintone. Among other findings, their results indicate that controlling for background characteristics such as childhood circumstances, education, and skills reduces the income gap between blacks and whites in their sample by half, but has a much lower effect on reducing the income gap between light and dark among those who identified as black.

Within the field of psychology, to understand the roots of skin tone bias, Smith-McLallen, Johnson, Dovidio, and Pearson [42] provide an excellent overview of what they term “color bias” in cultural associations. Color bias—which results from positive associations with the color white and negative associations with the color black, “independent of any explicit connection to race” (p. 48)—is pervasive. Anthropologist Margaret Mead [43] suggested that early humans’ “fear of the night, the dark, the unknown, and the unseen” could all be dispelled by the “light of a fire or of the moon or sun.” ([42] p. 48) In modern psychology research, evolving work suggests that human cognition may be affected in surprising ways by this seemingly primal bias. Ben Zeev et al.’s [21] work, for example, uses a two-study design to examine a phenomenon they call “skin tone memory bias.” In essence, their findings suggest that darker males who are identified as “more educated” (what the authors identify as “counter-stereotypic”) tend to be remembered as “whiter” than what they actually are. Ben Zeev et al., [21] suggest that we may be cognitively inclined to change our memory of skin tone “in an attempt to resolve an incompatible cognition in the direction of a stereotype [44]” (p. 7).

The emergence of dual process models of cognition, [2] as noted in the introduction, have produced breakthroughs in the understanding of the automatic and implicit processes through which biases of all types, including skin tone bias, may affect perception and decision-making. The use of the Implicit Association Test (IAT) [4] in conjunction with theoretical developments has also advanced our understanding of these processes. Briefly, the IAT is designed to measure associations between target categories (e.g., lighter skin, darker skin) and other positive vs. negative concepts or attributes (e.g., logic vs. chaos). Easier pairings result in faster responses, which are interpreted as more strongly associated in memory than slower responses. The pattern of effects is then examined for evidence of positive or negative bias with respect to the target categories [4]. There has been criticism of the IAT and the research that has resulted [45], but to date, the IAT has provided bias researchers with a methodology that has allowed a window into data collection on subconscious biases that individuals may hold with respect to different target groups. [1], [3], [6], [8], [9]

Although we do not provide a formal hypothesis here, the evidence reviewed above overwhelmingly suggests that darker skin tone likely leads to implicit bias, stereotyping, and more negative work outcomes for individuals. As a result, we expect that darker skin tone is negatively related to earnings.

### Joint effects of height and skin tone

Height has long been a metaphor for importance and power. [46] Within the psychology literature, the social esteem pathway provides some particularly interesting theoretical insights into what factors may be at play in linking height to measures of performance and career success. Judge and Cable, ([5], p. 429) define social esteem as “how positively one is evaluated or regarded by others in society.” Height is a factor that has historically been interpreted as a sign of power. As Judge and Cable ([5] p. 429) note, sociobiologists have long suggested that it was “evolutionarily advantageous for creatures to interpret height as power [47].” They further argue that visual perception and social norms have developed around the meaning of size and height. Indeed, prior research on visual perception has found that humans exhibit a basic perpetual bias whereby people judge an entity’s value or status, in part, by its size. [48], [49], [50] Indeed, height is likely used as a “heuristic for dominance,” ([51] p. 321) and many people find taller individuals more persuasive and convincing than shorter individuals. [52] Height is also correlated with intelligence; [53] researchers suggest that the underlying reason for this may be that both of these attributes may be markers of “system integrity.” [54] This idea is consistent with the social perceptions of height that we noted above.

A set of four large-sample studies conducted by Judge and Cable [5] makes a compelling argument that height is related to workplace success. Their theoretical model suggests that height results in social esteem and self-esteem, which in turn positively influence objective and subjective performance, which ultimately results in career success (and higher earnings). Their empirical findings focused on the relationship between height and career performance and success, which they argued were necessary to support in order to justify further investigation in future research into the mediating mechanisms. Interestingly, Judge and Cable’s [5] meta-analysis, in conjunction with earlier research on the importance of height as a predictor of leadership, performance, and other workplace outcomes, did not consider the issues of race or skin tone as potential moderating factors with respect to height. We contend, however, that height may operate differently for individuals with darker skin tones.

As noted above, prior research suggests that individuals with darker skin tones experience more bias, discrimination, and likely lower levels of earnings than individuals with lighter skin tones, and height has been considered to be synonymous with importance and power. [5] We contend that skin tone and height interact in complex ways that reflect the negative biases associated with individuals with darker skin tones, rather than the positive connotations normally associated with height. In particular, we expect that individuals who are taller and have darker skin tones will experience higher levels of discrimination and bias, which will lead to lower earnings over time. In part, this may be a result of the heavily negative stereotypes affecting individuals with darker skin tones—the perceptions of potential unpredictability and danger [21] might be exacerbated by individuals who are taller (i.e., more physically imposing). Prior research suggests that skin tone and physical attributes—like facial physiognomy—may each have an impact on the way that others evaluate individuals; [55] as observers perceive these characteristics, the combination of height and darker skin tone is likely to activating implicit biases and stereotypes. Of course, as individuals work in organizations over time, deep-level differences such as ability, personality, and experience [56] likely play a role in organizational advancement and income level, but we argue that taller individuals with darker skin may have an additional hurdle as they navigate their careers. Over time, we expect to see this manifest as a negative impact on the earnings of these individuals.

To sum up, the positive implicit and explicit associations with height have not been studied in conjunction with skin tone, but we assert that the positive associations with height may not hold true for individuals with darker skin tones. Indeed, individuals with darker skin tones who are taller may, in fact, experience more implicit bias and negative stereotyping as a result of their perceived physical power. This logic leads to the following hypothesis:

*Hypothesis 1* Height will moderate the negative relationship between skin tone (lightest to darkest) and earnings, such that the strength of the relationship is stronger (i.e., more negative) for taller individuals.

### Joint effects of gender, height, and skin tone

A third immediately noticeable physical attribute that has long been considered as an important antecedent of workplace outcomes (and is a source of potential bias and adverse impact) is gender. [57] In a recent meta-analysis, Koch, D’Mello, and Sackett [58] provide a review of the research on gender bias in workplace decisions, noting that both experimental and field study approaches have resulted in a substantial body of findings suggesting that gender has an impact on workplace decisions and outcomes, resulting in pervasive inequalities between men and women that seem hard to change. [59] This may be because gender is a “common cue for stereotypical thinking, with gender stereotypes being quickly and automatically activated [60], [61], [62], [63], [64], [65], [66].” ([58] p. 129) As Koch et al. [58] note, although stereotypes can be accurate, functional, and even helpful as a heuristic in the aggregate, [63], [64], [65] stereotypes can also result in critical errors in evaluation (i.e., bias) when ascribed to individual members of groups.

The three physical attributes discussed above (gender, height, and skin tone) likely present themselves at once to an observer; we propose that a complex interaction between the three attributes may result. While height may exacerbate the negative influence of darker skin tone on earnings overall, we expect that the ways in which skin tone and height may interact with males vs. females may be different. In other words, the interaction between skin tone and height on earnings may be different for each gender; we explicate below.

Women with darker skin tones may face less negative stereotyping (and resulting bias) than men with darker skin tones. Indeed, the body of research suggests that males with darker skin tones face heavily negative stereotypes. [66] For example, media outlets historically have portrayed darker-skinned men as aggressive, prone to criminal behavior, violent, and impulsive. [67] These stereotypes serve as the underlying justification for incarceration and profile-driven over-surveillance in black communities, among other issues. [67], [68], [69] We suggest that the physical presence of taller, darker men, in particular, may activate the most extreme negative stereotypes of darker-skinned men. In essence, skin tone together with height are two critical physical characteristics that activate observers’ negative stereotyping of the target individual, [21] particularly if that individual is male.

Although the above negative stereotyping certainly occurs for darker women, we expect stereotypes for women to be less affected by height. While we do not expect that the positive attributions associated with size and height [48], [49], [50] would hold for this particular group, or that height would necessarily be associated with attractiveness for this group, [70] the same level of intensity associated with the stereotype of physically imposing, darker men is likely not as salient. As we noted above, although over time deep-level differences such as personality, ability, and experience may help taller, darker males successfully navigate their careers, [56] we expect that the compounded impact over time of these heavily negative stereotypes for this group will result in a negative impact on earnings.

To conclude, while for women, the influence of darker skin tone on earnings may be less affected by height, men may be different. In particular, for men with darker skin tone, we expect that the negative relationship between skin tone and earnings will be stronger (i.e., more negative) for individuals who are taller.

*Hypothesis 2*. Height will moderate the relationship between skin tone and earnings differently for men vs. women; in particular, for men, the negative relationship between skin tone and earnings will be strengthened by height (i.e., more negative for taller darker skin tone males).

## Method

### Sample

We draw on the National Longitudinal Study of Youth (NLSY) 1997, a longitudinal and a nationally representative sample of individuals born between 1981 and 1985 who have been continuously followed since 1997. The NLSY 1997 survey is sponsored and directed by the U.S. Bureau of Labor Statistics and conducted by the National Opinion Research Center at the University of Chicago, with assistance from the Center for Human Resource Research at The Ohio State University. NLSY 1997 longitudinally measures home, school, and labor market outcomes of the respondents. The data for this study includes 134,745 individual-year observations from 1997 to 2011. To avoid issues on ethnic and skin tone variation among other minority populations such as the Latino population, and in line with Kreisman and Rangel, [27] we focus on black vs. white respondents in the NLSY 1997. It is important to note that we acknowledge that skin tone bias exists among other ethnic groups and countries across the world. While the inclusion of only black and white respondents in our analyses could limit the generalizability of our findings, based on Kreisman and Rangel [27], this approach provides more reliable estimates based on the availability of a critical mass of sample size for black and white respondents in NLSY 1997.

This filter resulted in 104,985 individual-year observations. Next, dropping observations with missing income data and skin-tone data resulted in a sample of 44,172 individual-year observations. After dropping observations with missing data on remaining variables in the model, the final sample consists of 31,356 individual-year observations across 4,340 individuals.

### Measures

#### Earnings

We used self-reported income to serve as the measure of earnings. Kreisman and Rangel [27] and other studies drawing on NLSY 1997 have used this measure. To reduce the influence of extreme observations, however, we used the natural log of inflation-adjusted total annual income from wages and salaries. We used consumer price index data from Bureau of Labor Statistics to adjust for inflation and express in 2011 dollars.

#### Skin tone

We measure skin tone based on the NLSY 2007 wave, when the interviewers coded skin tone using a skin tone scale. This skin tone scale was validated by Kreisman and Rangel. [27] Additionally, a similar scale was used in National Immigrant Survey conducted by NORC, the research body who also conducted NLSY 1997. The scale presents color images of human hands of similar size and shape, but with different skin tones. The scale ranges from 0 (lightest skin tone) to 10 (darkest skin tone). The interviewers were asked to memorize the scale before their interviews and then score skin tone accordingly. To ascertain the reliability of coding of skin tone by the interviewer, Akee and Yuksel [71] compare the interviewer ratings of skin tone with race level variations in skin tone in the CARDIA study using a reflectance spectrometer and found the comparable distribution of skin tone. In their additional analyses, Kreisman and Rangel [27] further establish the reliability of coding for skin tone in NLSY 1997.

#### Height

To measure height, we take the median height of the female (male) U.S. population between 2007 and 2010 from the National Center for Health Statistics and subtract a female (male) respondent’s self-reported height from the median female (male) height. As gains from height are relative to the gender-specific median height in the population, consideration of the deviation above or below the median height allows for normalization of individual height relative to height in the population.

#### Gender

We code gender of respondents as 0 = male, 1 = female, based on participants’ self-reported gender in the NLSY 1997.

#### Control variables

To reduce the effects of rival explanations, we control for *race* (white = 1, black = 0), *age* (in years), and whether the respondent is a *high school graduate* (= 1, else = 0). As *health status* could impact earnings, we include self-reported health status (5-point, Likert-type scale with 1 = poor health to 5 = excellent health). As *weight* may be a discriminatory and confounding factor, we control for weight in pounds. [26], [70] We also control for *marital status* (Never married = 0, Married = 1, Separate = 2, Divorced = 3, Widowed = 4) and *cognitive ability*, using the natural log of the score on the Armed Services Vocational Aptitude Battery (ASVAB). The raw ASVAB scores range from 0 to 100,000 with a mean of 58,440 and standard deviation of 27,872.23. We use the natural log of ASVAB score to reduce the variability of scores and to make them conform closer to a normal distribution. We include the natural log of *spousal income* from wages and salary in real terms. As the urban location of the respondent could affect the nature of perceptions of skin tone, we control for whether the respondent lives in a *metropolitan statistical area* (1 = MSA, else = 0). We also control for whether *mother* (= 1, else = 0) and/or *father* (= 1, else = 0) were *high school graduates*.

As real federal minimum wage sets the baseline income standards, we control for *real federal minimum wage* for each year. As a *recession* may negatively impact earnings, we include a dummy variable (1 = recession year; 0 = non-recession year) for recession using information on recession years as obtained from the National Bureau of Economic Research’s recession data. [72]

Finally, we control for *year* and *state* dummy variables. We also control for 34 occupation code dummy variables.

S1 Table lists the mean, standard deviation, and correlations based on casewise deletion.

## Results

We use pooled-OLS regression in Stata 15. Results indicate two findings of note that we expected from prior literature, but did not formally hypothesize. First, darker skin tone is negatively related to real income (β = -0.0216, *p* = 0.014, Table 1 Model 2). The average wage in the sample is $21,475.93 (s.d. = 21,133.97). For every one unit increase in darker skin tone (on a 10-point scale with 0 being the lightest and 10 being the darkest), annual real wages decline by $463.88 (= $21,475.93 × -0.0216). Considering this decline over a 40-year period (the average work-life span), at the average risk-free U.S. Treasury rate (from 1994 to 2016) of 4.90%, this amount translates to a lifetime loss of $54,687.85. Notably, this lifetime loss amount is multiplied when individuals are more than one unit darker than “lightest skin tone” (which was the lightest response on the skin tone scale)—so, for an individual rated a 10 on the scale (“darkest skin tone”), this amount would translate to a lifetime loss of $546,878.50, relative to an individual with the lightest skin tone (rated 0 = “lightest skin tone”). Second, findings indicate that height is positively related to income (β = 0.00576, *p* = 0.020, Table 1 Model 3). For each one-inch increase in height above the median population height within each gender, real income increases by $123.70 per year. Considering this effect over a 40-year period (again, assuming the U.S. Treasury rate of 4.90%), this amount translates to a lifetime gain of $14,583.27 for these individuals. Again, this lifetime gain amount is multiplied when individuals are more than one inch taller than the median population height within each gender; so an individual three inches taller than average might expect to make $43,749.81 more over the course of their lifetime.

**Table 1:** Pooled OLS regression results.

VARIABLES	(1) Log of total real income	(2) Log of total real income	(3) Log of total real income	(4) Log of total real income	(5) Log of total real income
Skin tone		-0.02*	-0.02*	-0.02*	-0.02
		(0.01)	(0.01)	(0.01)	(0.01)
Deviation height in inches			0.01*	0.01**	0.02**
			(0.00)	(0.00)	(0.00)
Skin tone × Deviation in height [H1]				-0.00**	-0.01**
				(0.00)	(0.00)
Female	-0.19**	-0.20**	-0.20**	-0.20**	-0.19**
	(0.02)	(0.02)	(0.02)	(0.02)	(0.02)
Female x Skin tone					0.00
					(0.01)
Female x Deviation in height					-0.01**
					(0.01)
Female x Skin tone x Deviation in height [H2]					0.01**
					(0.00)
Race (white)	0.16**	0.03	0.03	0.03	0.03
	(0.02)	(0.06)	(0.06)	(0.06)	(0.06)
Age	0.17**	0.17**	0.17**	0.17**	0.17**
	(0.01)	(0.01)	(0.01)	(0.01)	(0.01)
High school graduate	0.36**	0.36**	0.36**	0.36**	0.36**
	(0.02)	(0.02)	(0.02)	(0.02)	(0.02)
Health status	0.05**	0.05**	0.05**	0.05**	0.04**
	(0.01)	(0.01)	(0.01)	(0.01)	(0.01)
Weight in pounds	0.00**	0.00**	0.00**	0.00**	0.00**
	(0.00)	(0.00)	(0.00)	(0.00)	(0.00)
Married	0.06**	0.06**	0.06**	0.06**	0.06**
	(0.02)	(0.02)	(0.02)	(0.02)	(0.02)
Separated	-0.10	-0.10	-0.10	-0.10	-0.10
	(0.09)	(0.09)	(0.09)	(0.09)	(0.09)
Divorce	0.01	0.00	0.00	0.00	0.00
	(0.04)	(0.04)	(0.04)	(0.04)	(0.04)
Widowed	0.07	0.07	0.07	0.07	0.07
	(0.19)	(0.20)	(0.20)	(0.20)	(0.19)
Log of ASVAB score	0.07**	0.07**	0.07**	0.07**	0.07**
	(0.01)	(0.01)	(0.01)	(0.01)	(0.01)
Log of total spousal real income	0.02**	0.02**	0.02**	0.02**	0.02**
	(0.00)	(0.00)	(0.00)	(0.00)	(0.00)
Metropolitan Statistical Area	-0.02	-0.02	-0.02	-0.02	-0.02
	(0.03)	(0.03)	(0.03)	(0.03)	(0.03)
Dad is a high school grad	0.05*	0.05	0.05	0.04	0.04
	(0.02)	(0.02)	(0.02)	(0.02)	(0.02)
Mom is a high school grad	-0.04	-0.04	-0.04	-0.04	-0.04
	(0.03)	(0.03)	(0.03)	(0.03)	(0.03)
Real federal minimum wage	2.53**	2.51**	2.53**	2.53**	2.47**
	(0.51)	(0.51)	(0.51)	(0.51)	(0.51)
Recession years	-0.58**	-0.57**	-0.58**	-0.58**	-0.56**
	(0.20)	(0.20)	(0.20)	(0.20)	(0.20)
Constant	-14.43**	-14.16**	-14.27**	-14.31**	-13.86**
	(3.61)	(3.615)	(3.62)	(3.61)	(3.61)
Individual-year observations	31,356	31,356	31,356	31,356	31,356
F-Stat	182.03	180.40	178.81	177.92	174.88
Log likelihood	-49213.28	-49209.34	-49206.51	-49200.46	-49193.31

Notes.

Robust standard errors in parenthesis.

Year dummies, Occupation code dummies, and State of residence dummies are included in all the models.

** *p* < 0.01,

* *p* < 0.05 (two-tailed)

In Hypothesis 1, we proposed that the relationship between skin tone and real income will be stronger (i.e., more negative) for individuals who are taller. Results support this hypothesis (β = -0.00298, *p* = 0.004; Table 1, Model 4); or, with a one-inch increase in height *and* a one-unit increase in darkness of skin tone relative to lightest skin tone, income declines by an additional $64.00, for a total loss in one year of $527.88 (significantly more than the $463.88 loss for a one-unit increase in darkness of skin tone without the increase in height). This translates to an additional lifetime loss of $7,545.10, or $75,541 for individuals who are darkest on the skin tone scale (again, this would be in addition to the loss of $546,878.50 for individuals who are darkest on the skin tone scale without the added increase in height; so the total lifetime loss for the darkest individuals who are one inch taller than average would be $622,419.50 relative to those with lightest skin tone).

**Fig 1:**
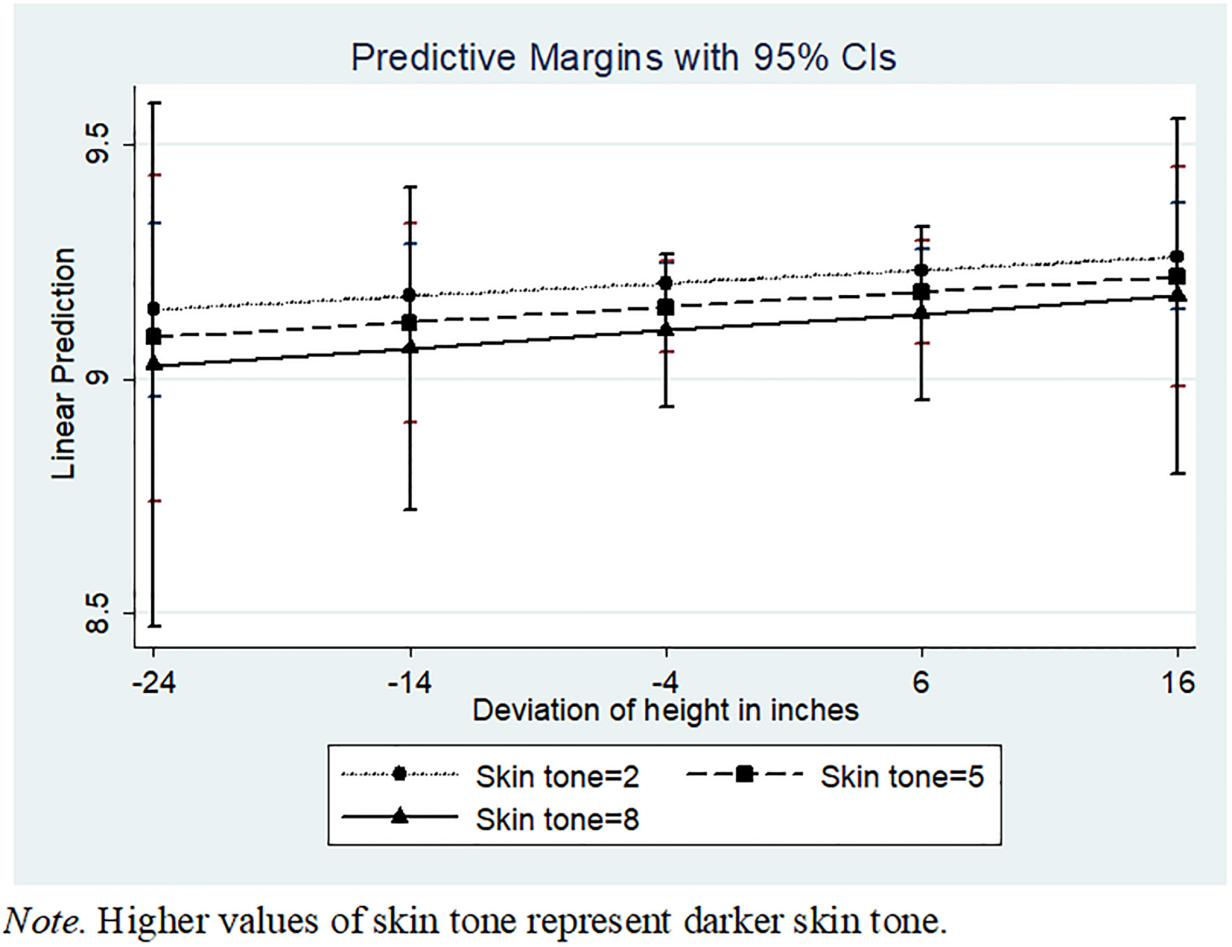
Three-way interaction between skin tone, height, and gender. Graphed lines indicate impact of skin tone and height on earnings for women.

**Fig 2:**
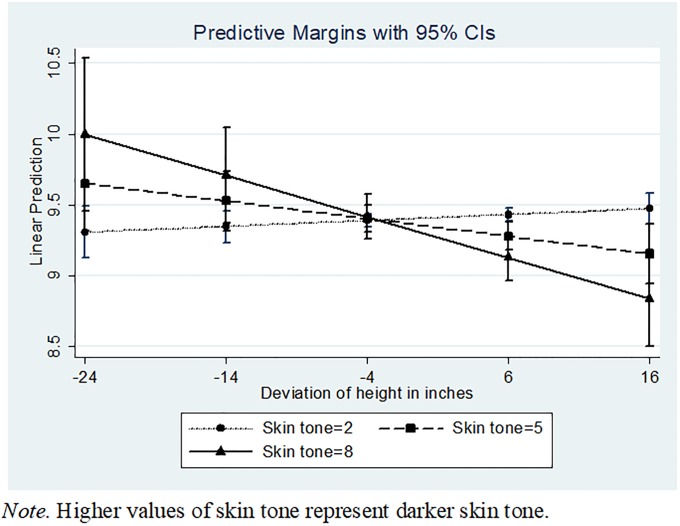
Three-way interaction between skin tone, height, and gender. Graphed lines indicate impact of skin tone and height on earnings for men.

Table 1 Model 5 presents the three-way interaction of skin tone × deviation in height × gender, proposed in Hypothesis 2. As hypothesized, we find that for males, taller and darker attributes lead to lower income (β = -0.00553, *p* = 0.000, Table 1 Model 5). In contrast, taller females with darker skin tone actually earn higher income (β = 0.00568, *p* = 0.007). Figs 1 and 2 show the marginal effects of skin tone and height for females and males, respectively. For black males, for each unit increase in skin tone and a one-inch increase in height above the median height, the lifetime “pay cut” translates to an additional $118.76 per year, or (assuming a 4.9% return and a 40-year career) a lifetime loss of an additional $14,000.88. This pay cut is most severe for black males who are darkest on the skin tone scale, with an additional lifetime loss of $140,008.80 (again, this would be in addition to the baseline loss of $546,878.50; the total lifetime loss for black males who are darkest on the skin tone scale and one inch taller than average would be $686,887.30, relative to those with lightest skin tone).

### Additional Analyses

#### Robustness tests

Though we control for the state of residence in the above analysis, we perform an additional robustness test by subtracting the state median household income from individual income. Systematic variations across states in economic activity and growth could conflate individual income. The state median household income data was obtained from the US Census Small Area Income and Poverty Estimates. Table 2 Model 1 shows the results of this analysis; we find that the direction and significance of predictors are consistent with our main results.

**Table 2:** Robustness tests.

VARIABLES	(1) Deviation in income from state-level median household income	(2) Log of total real income	(3) Log of total real income
Skin tone	-148.50	-0.02**	-0.02
	(98.05)	(0.01)	(0.01)
Deviation height in inches	329.66**	0.02**	0.02**
	(53.71)	(0.00)	(0.00)
Skin tone × Deviation in height [H1]	-76.79**	-0.01**	-0.01**
	(14.50)	(0.00)	(0.00)
Female	-3,836.62**	-0.19**	-0.19**
	(255.08)	(0.02)	(0.02)
Female x Skin tone	247.35**	0.00	0.00
	(65.37)	(0.01)	(0.01)
Female x Deviation in height	-316.85**	-0.01*	-0.01**
	(71.02)	(0.01)	(0.00)
Female x Skin tone x Deviation in height [H2]	84.10**	0.01**	0.01**
	(20.14)	(0.00)	(0.00)
Race (white)	1,928.77**		0.03
	(628.15)		(0.05)
Age	2,547.60**	0.17**	0.17**
	(76.26)	(0.01)	(0.01)
High school graduate	1,612.52**	0.36**	0.36**
	(228.46)	(0.02)	(0.02)
Health status	1,203.85**	0.04**	0.04**
	(106.74)	(0.01)	(0.01)
Weight in pounds	4.26	0.00**	0.00**
	(2.43)	(0.00)	(0.00)
Married	4,566.37**	0.06**	0.06**
	(375.68)	(0.02)	(0.02)
Separated	-545.65	-0.10	-0.11
	(1,045.49)	(0.08)	(0.09)
Divorce	-927.69	0.00	0.01
	(655.30)	(0.05)	(0.04)
Widowed	-6,842.43*	0.07	0.06
	(3,442.37)	(0.32)	(0.19)
Log of ASVAB score	870.83**	0.07**	0.06**
	(105.35)	(0.01)	(0.01)
Log of total spousal real income	120.03**	0.02**	0.02**
	(32.35)	(0.00)	(0.00)
Metropolitan Statistical Area	-388.18	-0.02	-0.01
	(273.24)	(0.03)	(0.03)
Dad is a high school grad	1,090.27**	0.04*	0.04
	(269.13)	(0.02)	(0.02)
Mom is a high school grad	496.57	-0.04	-0.04
	(298.09)	(0.02)	(0.02)
Real federal minimum wage	12,408.98	2.45**	2.66**
	(6,391.61)	(0.49)	(0.51)
Recession years	-3,555.33	-0.55**	-0.63**
	(2,656.19)	(0.19)	(0.19)
Share of Black population in the respondent’s state of residence			-14.72**
			(2.08)
Constant	-180,701.34**	-13.73**	-11.33**
	(44,500.96)	(3.44)	(3.63)
Estimation methods	Pooled-OLS	Mixed effects Model	Pooled-OLS
Observations	31,356	31,356	31,356
R-squared	0.470		0.380

Notes.

Robust standard errors in parenthesis for Model 1 and 3.

Skin tone is nested under race for the mixed effects model (Model 2).

Year dummies, Occupation code dummies, and State of residence dummies included in all the models.

** *p* < 0.01,

* *p* < 0.05

We also test our results using a multi-level linear regression model with skin color nested within race (using *mixed* routine in Stata 15). Table 2 Model 2 shows the results of the analysis, which are consistent with the original model.

Last, varying racial prejudice at the state level could confound with lower income associated with darker skin tone. We include the share of black population in a state for each survey year as an additional control. The data were obtained from the US Census Bureau’s population estimates program. We find that our original results are robust, and the higher share of black population in a state is associated with lower income (Table 2 Model 3).

#### Categorical measure of skin tone

We use a continuous measure of skin tone in the main analysis. We also consider whether the results might change under a categorical operationalization of skin tone: light black (skin tone 1 to 5), medium black (skin tone 6 and 7), and dark black (skin tone 8 to 10). Results using this categorical operationalization showed consistent findings (see Table 3).

**Table 3:** Additional test with “blurred” categorical color line—Pooled OLS regression results.

VARIABLES	(1) Log of total real income	(2) Log of total real income	(3) Log of total real income
Light black (skin tone 1 to 5)	-0.09**	-0.08**	-0.08
	(0.03)	(0.03)	(0.05)
Medium black (skin tone 6 to 7)	-0.21**	-0.20**	-0.22**
	(0.03)	(0.03)	(0.05)
Dark black (skin tone 8 to 10)	-0.18**	-0.14**	-0.14**
	(0.04)	(0.04)	(0.05)
Female	-0.20**	-0.20**	-0.20**
	(0.02)	(0.02)	(0.02)
Deviation height in inches	0.01*	0.01**	0.02**
	(0.00)	(0.00)	(0.00)
Light black × Deviation in height [H1]		-0.01	-0.04*
		(0.01)	(0.02)
Medium black × Deviation in height [H1]		-0.01	-0.03
		(0.01)	(0.01)
Dark black × Deviation in height [H1]		-0.03**	-0.04**
		(0.01)	(0.02)
Female x Deviation in height			-0.01**
			(0.00)
Female x Light black			0.01
			(0.06)
Female x Medium black			0.04
			(0.06)
Female x Dark black			-0.00
			(0.07)
Female x Light black x Deviation in height [H2]			0.05**
			(0.02)
Female x Medium black x Deviation in height [H2]			0.04
			(0.02)
Female x Dark black x Deviation in height [H2]			0.02
			(0.02)
Age	0.17**	0.17**	0.17**
	(0.01)	(0.01)	(0.01)
High school graduate	0.35**	0.36**	0.36**
	(0.02)	(0.02)	(0.02)
Health status	0.05**	0.04**	0.04**
	(0.01)	(0.01)	(0.01)
Weight in pounds	0.00**	0.00**	0.00**
	(0.00)	(0.00)	(0.00)
Married	0.06**	0.06**	0.06**
	(0.02)	(0.02)	(0.02)
Separated	-0.09	-0.10	-0.10
	(0.09)	(0.09)	(0.09)
Divorce	0.00	0.00	0.00
	(0.04)	(0.04)	(0.04)
Widowed	0.06	0.06	0.07
	(0.20)	(0.20)	(0.19)
Log of ASVAB score	0.07**	0.07**	0.07**
	(0.01)	(0.01)	(0.01)
Log of total spousal real income	0.02**	0.02**	0.02**
	(0.00)	(0.00)	(0.00)
Metropolitan Statistical Area	-0.02	-0.02	-0.02
	(0.03)	(0.03)	(0.03)
Dad is a high school grad	0.05	0.04	0.04
	(0.02)	(0.02)	(0.02)
Mom is a high school grad	-0.04	-0.04	-0.04
	(0.02)	(0.02)	(0.02)
Real federal minimum wage	2.52**	2.52**	2.47**
	(0.51)	(0.51)	(0.51)
Recession years	-0.58**	-0.58**	-0.56**
	(0.20)	(0.20)	(0.20)
Constant	-14.22**	-14.20**	-13.84**
	(3.62)	(3.62)	(3.62)
Observations	31,356	31,356	31,356
R-squared	0.38	0.38	0.38
F-test	177.26	173.49	165.60

Notes.

Robust standard errors in parenthesis.

Year dummies, Occupation code dummies, and State of residence dummies included in all the model.

** *p* < 0.01,

* *p* < 0.05

#### Mitigating effects of education

In the human capital investment literature, [73], [74] a framework in economics, the expectation is that human capital investments (such as educational attainment, work experience, training, etc.) would result in higher returns to the individual and, for the purposes of this study, greater earnings. Interestingly, prior research findings suggest that women and minorities “do not get the same career return on their human capital investments as do majority group members with the same level of human capital investment.” ([13], p. 749) Although individuals with higher education levels likely earn more, this may be different for males with darker skin tones. We, therefore, examine whether the effects of the interaction between skin tone and deviation in height for males with darker skin tones are different depending on their level of education; to do this, we create subgroups of individuals with and without a high school education.

As reported in Table 4, we find that for the group that received education beyond high school, males with darker skin tones experience a greater loss of income. In other words, the effects of skin tone for taller, darker males worsens for those with a high school education or above. The interpretation is as follows: total effect for male and female is [(Skin tone × Deviation in height) + (Gender × Skin tone × Deviation in height)], for males by substituting zero, the effect is the estimate for Skin tone × Deviation in height. Therefore, for each one-inch increase in height and one one-unit increase in the darkness of skin tone, taller, darker males with a high school education realize an additional yearly income cut of $123.48 (= $21,475.93 × 0.00575), translating to a lifetime loss of $14,557.33 (assuming the 4.9% return rate). Multiplying this out, high-school graduate (or higher) males who are one inch taller than average and darkest on the skin tone scale would experience a lifetime loss of $145,573.30 relative to those with the lightest skin tone, in addition to the baseline loss of $546,878.50 (total lifetime loss amount would equal $692,451.80) relative to those with the lightest skin tone. In sum, while darker and taller males are likely making more money overall because of their human capital investment in education, males who are darker and taller continue to realize lower earnings than their counterparts with lighter skin tones.

**Table 4:** Sub-group analysis by education, ability, and income.

	Log of total real income				
VARIABLES	Education		Ability		High income
	No High school grad	High school grad	1st quantile of ASVAB scores	4th quantile of ASVAB scores	Total annual income > = 75th percentile
Skin tone	-0.02	-0.02	-0.02	-0.06**	-0.00
	(0.03)	(0.01)	(0.02)	(0.02)	(0.00)
Deviation height in inches	0.01	0.02**	0.02*	0.02**	0.01**
	(0.01)	(0.00)	(0.01)	(0.01)	(0.00)
Skin tone × Deviation in height [H1]	-0.01	-0.01**	-0.00	-0.03**	-0.00**
	(0.00)	(0.00)	(0.00)	(0.01)	(0.00)
Female	-0.27**	-0.18**	-0.38**	-0.06	-0.07**
	(0.05)	(0.02)	(0.05)	(0.04)	(0.01)
Female x Skin tone	-0.02	0.00	0.00	0.03	0.00
	(0.02)	(0.01)	(0.01)	(0.02)	(0.00)
Female x Deviation in height	0.00	-0.01**	0.00	-0.02*	-0.01**
	(0.01)	(0.01)	(0.01)	(0.01)	(0.00)
Female x Skin tone x Deviation in height [H2]	0.01	0.00*	-0.00	0.02*	0.00**
	(0.01)	(0.00)	(0.00)	(0.01)	(0.00)
Race (white)	0.16	0.01	0.26*	-0.44**	0.04
	(0.19)	(0.06)	(0.10)	(0.12)	(0.03)
Age	0.23**	0.16**	0.14**	0.21**	0.04**
	(0.02)	(0.01)	(0.01)	(0.01)	(0.00)
High school graduate			0.39**	0.24**	0.02
			(0.04)	(0.06)	(0.02)
Health status	0.02	0.06**	0.03	0.02	0.02**
	(0.02)	(0.01)	(0.02)	(0.02)	(0.00)
Weight in pounds	0.00**	0.00**	0.00**	0.00	-0.00
	(0.00)	(0.00)	(0.00)	(0.00)	(0.00)
Married	0.29**	0.05*	0.15**	0.08	0.07**
	(0.08)	(0.02)	(0.05)	(0.04)	(0.01)
Separated	-0.11	-0.10	-0.14	-0.26	0.03
	(0.21)	(0.10)	(0.15)	(0.27)	(0.03)
Divorce	-0.08	0.02	0.11	-0.09	-0.05**
	(0.17)	(0.04)	(0.09)	(0.15)	(0.02)
Widowed	0.28	0.08	-0.13	0.22	0.08**
	(0.17)	(0.31)	(0.31)	(0.21)	(0.02)
Log of ASVAB score	0.10**	0.05**			0.02**
	(0.03)	(0.01)			(0.01)
Log of total spousal real income	0.03**	0.02**	0.02**	0.01**	-0.00**
	(0.01)	(0.00)	(0.00)	(0.00)	(0.00)
Metropolitan Statistical Area	-0.03	0.01	0.03	-0.03	0.01
	(0.06)	(0.03)	(0.06)	(0.05)	(0.02)
Dad is a high school grad	0.00	0.07**	0.11**	-0.04	0.04**
	(0.05)	(0.03)	(0.04)	(0.06)	(0.01)
Mom is a high school grad	-0.07	-0.02	0.08	-0.11	0.01
	(0.05)	(0.03)	(0.04)	(0.07)	(0.01)
Real federal minimum wage	-3.72*	3.01**	3.14*	1.37	-0.23*
	(1.59)	(0.69)	(1.22)	(0.96)	(0.11)
Recession years	1.80**	-0.78**	-0.83	-0.11	0.09
	(0.64)	(0.25)	(0.46)	(0.37)	(0.05)
Constant	28.42*	-16.98**	-17.67*	-5.26	11.10**
	(11.11)	(4.95)	(8.62)	(6.71)	(0.72)
Observations	5,289	26,067	7,844	7,833	7,828
R-squared	0.37	0.31	0.30	0.47	0.24

Notes.

Robust standard errors in parenthesis.

Year dummies, Occupation code dummies, and State of residence dummies included in all the model.

** *p* < 0.01,

* *p* < 0.05

#### Mitigating effects of cognitive ability

Based on the human capital investment arguments noted above, it is possible that individuals with higher cognitive ability may be able to avoid the losses associated with skin tone and height. In order to examine this, we split the sample into individuals scoring below the 1^st^ quantile and those scoring above 4^th^ quantile, based on raw ASVAB scores available in the data. As reported in Table 4, the total effect for taller males with darker skin tones with ASVAB score in the fourth quantile: [(Skin tone × Deviation in height) + (Gender x Skin tone x Deviation in height)], or -0.0276, translates into $592.74 of lower yearly income. With a one-inch increase in height above the median and a one-unit increase in darker skin tone, which would equal a lifetime loss of $69,879.45 (assuming a 4.9% return rate and a 40-year work span; this would be a lifetime loss of $698,794.50 for a 10-unit increase in the darkness of skin tone). This result suggests that taller, darker males with higher cognitive ability do, in fact, realize a significant income loss with increasing height and darker skin tone—the losses in dollar amount are greatest for this group, perhaps because the income potential is highest for this group, as well.

#### Higher income strata

While education and cognitive ability seem to increase the losses with respect to income levels for taller males with darker skin tone, these proxies for human capital may not represent cumulative life experiences. As a proxy for a combination of these multiple factors, we assess whether individuals with income above the 75^th^ percentile have similar patterns of effects. As presented in Table 4, we find that for darker males who, on average, have income above the 75^th^ percentile in the sample, for each unit increase in darker skin tone and each one-inch increase in height above the median, the lifetime total decline in earnings would be $4,227.62 ($35.86 per year, over 40 years, with a 4.9% return rate); again, for a 10-unit increase in darkness of skin tone, the losses would add up to approximately $422,762. The findings of the exploratory analyses presented above appear to support and extend prior findings that white males are likely to advance further and faster in their careers than minorities and women. [13], [75]

## Discussion

Drawing on implicit bias and stereotyping theories, we tested the joint effects of skin tone, height, and gender to assess whether the combination of these characteristics influences earnings, as measured by income. This is the first study to our knowledge to use multiple demographic variables, in combination with observer-rated skin tone, to predict earnings reported in a longitudinal sample. The findings of this study suggest that darker skin tone is negatively related to income, and that this effect is moderated by both height and gender, such that taller, darker males experience the most negative impact on their career success. These findings have significant implications for the human capital and compensation literature. [74], [76], [77], [78], [79], [80] Additionally, the findings contribute to the literature on skin tone bias that has been developing for decades in various fields. [23], [29], [30], [31], [32], [33], [34], [81]

While different theoretical perspectives could provide reasonable explanations for the effects found in this study, skin tone may be the observable physical characteristic, after controlling for a variety of individual and geographic factors, with the most negative impact on earnings (at least for the individuals in their 30s in the sample). Surprisingly, this lifetime “pay cut” in earnings is greater for those with higher education, higher cognitive ability (as operationalized by higher ASVAB scores), and even for those in the higher income strata (> 75%). In other words, while improving human capital factors may have led to higher income for taller darker males (relative to those with lower education, lower cognitive ability, or lower income strata), within more educated, higher cognitive ability, or higher income groups the income gaps persist. In other words,—human capital endowments do not seem to close income gaps for taller, darker males in these groups. What might explain this set of findings?

The findings seem to support theories in psychology over those in economics. From human capital theory in economics, greater education levels or higher cognitive ability could reduce asymmetric information, allowing individuals to overcome labor market discrimination through education and ability. The effects of discrimination are expected to be lower when other human capital signals (e.g., education and ability) are present. However, the results of the current study show that for the taller, darker males included in this study, income loss seems to increase in the presence of these signals. Psychological explanations (e.g., implicit bias and stereotyping) may be better suited to shed light on this phenomenon than economic explanations. In particular, the strongly negative stereotypes associated with darker black men may be exacerbated by agentic activity (i.e., stereotypically male behavior) in professional settings. Individuals who complete high school and who have higher levels of cognitive ability may, in fact, be perceived by others as acting in more agentic ways, resulting paradoxically in even more negative stereotyping. These individuals may be implicitly perceived as threats to the system; the resulting effect may be that they experience adverse impact with respect to their income levels.

Once again, these findings seem to support the extant research on bias and discrimination in the workplace; this work points to the fact that women and racial/ethnic minorities seem to experience the workplace in ways that are subjectively and objectively fundamentally different from their white male counterparts, [13], [82], [83] which ultimately leads to lower levels of earnings. Indeed, even with an infusion of human capital investment, middle-class minority workers’ experiences at work seem to echo this study’s findings. In a qualitative examination of several hundred cases of workplace discrimination against African American workers, Roscigno, Williams, & Byron [84] find that middle-class African Americans experience higher levels of firing discrimination, mobility-based discrimination, and day-to-day racial harassment. Similarly, Browne and Misra [85] find that human capital investments did not provide the same returns for black men and women as they did for white men and women; there were differences in returns within race by gender, as well. Importantly, our analysis reveals that even when the race is considered as a control variable, individuals with darker skin tones experience this negative impact on their earnings.

One unexpected finding in this study was that women in the sample, regardless of skin tone, seem to exhibit a positive relationship between height and income (as shown in Fig 1). While we had expected darker, taller males to be more affected by bias and stereotyping than their female counterparts (Fig 2), we did not expect for there to be essentially no differences for women with different skin tones. Prior literature on intersectionality and the ways in which women are affected by bias, discrimination, and stereotypes would suggest that women are disadvantaged in the workplace in numerous ways. [86], [87], [88] This study’s additional consideration of height may be an important consideration in future research. Theoretically, it is possible that the hypothesized relationships between height and social esteem and self-esteem that Judge and Cable [5] propose might lead taller women with darker skin tones to perform confidently and conscientiously at work, ultimately resulting in higher earnings. It seems unlikely, however, that simply being taller would serve as a way for a woman with darker skin tone to circumvent centuries of entrenched bias and discrimination; there are likely other mediators and/or moderators in play that should be examined in future research.

It is important to note that these findings provide only preliminary evidence of the complex triple impact of skin tone, height, and gender on earnings, despite the fact that the sample is from a large-scale study that is representative of U.S. millennials (NLSY1997). The results are especially illuminating, given that for this generation the general social narrative is that skin tone and racial boundaries are blurring. [89] The pattern of results suggests that strong negative stereotypes continue to impact negatively the youngest generation in the workforce. Our findings also shed light on the disturbing trend indicated in ([10], p. 62): that “Black males’ proportion of the labor force in 1970 was greater than in 2010… [they] are nine times more likely than White men to be incarcerated and more likely to be out of the labor force even when not incarcerated.” Unfortunately, it seems that implicit bias and stereotyping as a result of both race/ethnicity and skin tone continues to affect all kinds of decisions and institutions in our modern era.

The findings must be interpreted in the light of this study’s limitations. First, although we draw on NLSY 1997, one of the most widely used studies (https://nlsinfo.org/bibliography-start), and control for a variety factors, we are unable to tap into the rich micro-dynamics with respect to specific perceptions of skin tone, height, and gender. Despite this, it is important to note that like Byars-Winston et al., [10] this study is an attempt to provide a broader understanding of some of the trends associated with these individual characteristics that may be affecting individuals’ ability to attain objective career success. Future experimental studies could further shed light on potential mediating or moderating mechanisms. Second, while causation is not claimed nor implied, all three physical characteristics examined in this study are stable features during one’s adult lifespan, thereby limiting simultaneity between earnings and these characteristics. In closing, the constellation of darker skin tone, taller height, and gender (male) appears to have a significant negative influence on earnings in the U.S. We hope that our findings spur continued interest in implicit bias and stereotyping theories to promote future studies on the pathways through which “surface-level” individual characteristics likely influence important career outcomes such as earnings.

## Supporting information

S1 Table Pairwise correlations and descriptive statistics. (DOCX)
