# Supplementary material for: The effects of skin tone, height, and gender on earnings
Source: PLoS One. 2018 Jan 2;13(1):e0190640. doi: 10.1371/journal.pone.0190640 (PMC5749813; doi:10.1371/journal.pone.0190640)
Supplement: S1 Table — (DOCX) [file pone.0190640.s001.docx]

**S1 Table. Pairwise Correlations and descriptive statistics**

|  | Description | Mean | Std dev | Min | Max | 1 | 2 | 3 | 4 | 5 | 6 | 7 |
| --- | --- | --- | --- | --- | --- | --- | --- | --- | --- | --- | --- | --- |
| 1 | Log of total real income | 9.34 | 1.47 | 0 | 11.89 | 1.00 |  |  |  |  |  |  |
| 2 | Age | 23.31 | 3.73 | 16 | 32 | 0.55 | 1.00 |  |  |  |  |  |
| 3 | High school graduate | 0.83 | 0.37 | 0 | 1 | 0.31 | 0.33 | 1.00 |  |  |  |  |
| 4 | Health status | 3.95 | 0.89 | 1 | 5 | 0.00 | -0.10 | 0.05 | 1.00 |  |  |  |
| 5 | Weight in pounds | 172.73 | 44.42 | 1 | 450 | 0.17 | 0.21 | 0.06 | -0.16 | 1.00 |  |  |
| 6 | Marital status-married | 0.20 | 0.40 | 0 | 1 | 0.26 | 0.38 | 0.11 | -0.01 | 0.10 | 1.00 |  |
| 7 | Marital status-separate | 0.01 | 0.09 | 0 | 1 | 0.01 | 0.05 | -0.02 | -0.04 | ***0.00*** | -0.04 | 1.00 |
| 8 | Marital status-divorced | 0.02 | 0.15 | 0 | 1 | 0.06 | 0.13 | 0.01 | -0.03 | 0.02 | -0.07 | -0.01 |
| 9 | Marital status-widowed | 0.00 | 0.02 | 0 | 1 | ***0.01*** | 0.02 | -0.01 | -0.02 | ***-0.01*** | -0.01 | ***0.00*** |
| 10 | Log of ASVAB score | 10.70 | 0.91 | 0 | 11.51 | 0.07 | -0.03 | 0.21 | 0.13 | -0.07 | 0.04 | -0.03 |
| 11 | Log of total spousal real income | 2.67 | 4.49 | 0 | 12.91 | 0.27 | 0.38 | 0.13 | -0.04 | 0.06 | 0.61 | 0.04 |
| 12 | Metropolitan Statistical Area | 0.90 | 0.30 | 0 | 1 | 0.11 | 0.18 | 0.11 | 0.01 | ***0.00*** | 0.03 | ***0.00*** |
| 13 | Dad is high school grad | 0.88 | 0.33 | 0 | 1 | 0.04 | ***0.00*** | 0.15 | 0.09 | -0.04 | ***0.01*** | -0.03 |
| 14 | Mom is high school grad | 0.90 | 0.31 | 0 | 1 | 0.03 | ***0.00*** | 0.16 | 0.08 | -0.03 | ***0.01*** | -0.02 |
| 15 | Real Federal minimum wage | 6.66 | 0.57 | 5.75 | 7.60 | 0.12 | 0.40 | -0.05 | -0.05 | 0.07 | 0.16 | 0.03 |
| 16 | Recession year | 0.25 | 0.43 | 0 | 1 | 0.08 | 0.12 | 0.01 | -0.02 | 0.03 | 0.05 | 0.02 |
| 17 | Race (white) | 0.80 | 0.40 | 0 | 1 | 0.06 | -0.05 | 0.01 | 0.07 | -0.10 | 0.10 | -0.02 |
| 18 | Skin tone (0=white; 10 = dark black) | 1.23 | 2.61 | 0 | 10 | -0.06 | 0.05 | -0.02 | -0.06 | 0.10 | -0.09 | 0.02 |
| 19 | Deviation of gender specific average Height in inches | 1.30 | 2.95 | -33.5 | 26.5 | 0.05 | 0.01 | 0.05 | 0.05 | 0.32 | 0.03 | -0.01 |
| 20 | Female | 0.48 | 0.50 | 0 | 1 | -0.09 | ***0.00*** | 0.08 | -0.07 | -0.36 | 0.04 | 0.02 |

*Notes.*

Descriptive statistics: N=31,356 ID × year pooled observations across 4,340 individuals

Correlations in bold and italics are not significant at 0.10 or below (two-tailed), the remaining correlations are significant at .10 level (two-tailed)

Year, state, and occupation codes are excluded for the summary statistics

S1 Table. (*continued*)

|  | Description | 8 | 9 | 10 | 11 | 12 | 13 | 14 | 15 | 16 | 17 | 18 | 19 | 20 |
| --- | --- | --- | --- | --- | --- | --- | --- | --- | --- | --- | --- | --- | --- | --- |
| 8 | Marital status-divorced | 1.00 |  |  |  |  |  |  |  |  |  |  |  |  |
| 9 | Marital status-widowed | ***0.00*** | 1.00 |  |  |  |  |  |  |  |  |  |  |  |
| 10 | Log of ASVAB score | -0.03 | -0.02 | 1.00 |  |  |  |  |  |  |  |  |  |  |
| 11 | Log of total spousal real income | 0.03 | ***0.01*** | 0.03 | 1.00 |  |  |  |  |  |  |  |  |  |
| 12 | Metropolitan Statistical Area | ***0.01*** | ***0.00*** | 0.05 | 0.05 | 1.00 |  |  |  |  |  |  |  |  |
| 13 | Dad is high school grad | -0.04 | -0.02 | 0.22 | ***0.00*** | 0.07 | 1.00 |  |  |  |  |  |  |  |
| 14 | Mom is high school grad | -0.02 | ***0.01*** | 0.23 | ***0.01*** | 0.07 | 0.31 | 1.00 |  |  |  |  |  |  |
| 15 | Real Federal minimum wage | 0.07 | 0.01 | ***-0.01*** | 0.15 | ***0.01*** | ***0.00*** | ***-0.01*** | 1.00 |  |  |  |  |  |
| 16 | Recession year | 0.01 | ***0.00*** | ***0.00*** | 0.07 | ***0.01*** | ***0.00*** | ***-0.01*** | 0.36 | 1.00 |  |  |  |  |
| 17 | Race (white) | 0.03 | ***0.00*** | 0.31 | 0.10 | -0.06 | 0.09 | 0.10 | -0.01 | -0.01 | 1.00 |  |  |  |
| 18 | Skin tone (0=white; 10 = dark black) | -0.03 | ***0.00*** | -0.31 | -0.10 | 0.05 | -0.10 | -0.10 | 0.01 | 0.01 | -0.94 | 1.00 |  |  |
| 19 | Deviation of gender specific average height in inches | ***0.00*** | ***-0.01*** | 0.07 | 0.02 | ***0.00*** | 0.05 | 0.06 | ***-0.01*** | ***0.00*** | 0.06 | -0.05 | 1.00 |  |
| 20 | Female | 0.03 | 0.01 | 0.08 | 0.12 | 0.04 | 0.02 | ***0.00*** | ***0.00*** | ***0.01*** | -0.08 | 0.06 | -0.07 | 1.00 |

*Notes.*

Descriptive statistics: N = 31,356 ID × year pooled observations across 4,340 individuals

Correlations in bold and italics are not significant at 0.10 or below (two-tailed), the remaining correlations are significant at .10 level (two-tailed)

Year, state, and occupation codes are excluded for the summary statistics
